# Target Mechanism of Iturinic Lipopeptide on Differential Expression Patterns of Defense-Related Genes against *Colletotrichum acutatum* in Pepper

**DOI:** 10.3390/plants11091267

**Published:** 2022-05-09

**Authors:** Joon Seong Park, Gwang Rok Ryu, Beom Ryong Kang

**Affiliations:** 1 Gwangju Metropolitan City Agricultural Extension Center, Gwangju Metropolitan City 61945, Korea; acblue20@korea.kr; 2 Department of Agricultural & Biological Chemistry, Chonnam National University, Gwangju 61186, Korea; fbrhkdfhr@naver.com; 3 Institute of Environmentally-Friendly Agriculture, Chonnam National University, Gwangju 61186, Korea

**Keywords:** antifungal activity, *Bacillus subtilis* KB21, cyclic lipopeptides, pepper anthracnose

## Abstract

*Bacillus subtilis* KB21 is an isolate with broad spectrum antifungal activity against plant pathogenic fungi. Our aim was to produce and purify antifungal lipopeptides via fermentation using *B. subtilis* KB21 and verify their antifungal mechanism against pepper anthracnose. When the KB21 strain was cultured in tryptic soy broth medium, the antifungal activity against pepper anthracnose correlated with biosurfactant production. However, there was no antifungal activity when cultured in Luria-Bertani medium. KB21 filtrates showed the highest degree of inhibition of mycelia (91.1%) and spore germination (98.9%) of *Colletotrichum acutatum* via increases in the biosurfactant levels. Using liquid chromatography-mass spectrometry (LC-MS) and LC-tandem MS (LC-MS/MS) analyses, the component with antifungal activity in the fermentation medium of the KB21 strain was determined to be the cyclic lipopeptide (CLP) antibiotic, iturin A. When the iturin fractions were applied to pepper fruits inoculated with conidia of *C. acutatum*, the lesion diameter and hyphal growth on the fruit were significantly suppressed. In addition, iturin CLP elevated the gene expression of *PAL*, *LOX*, and *GLU* in the treatments both with and without following fungal pathogens. Overall, the results of this study show that iturin CLPs from *B. subtilis* KB21 may be potential biological control agents for plant fungal diseases.

## 1. Introduction

Pepper (*Capsicum annuum* L.) is an economically important crop in many countries but is susceptible to various diseases. Pathogens of the genus *Colletotrichum* can cause severe yield loss. The diseases caused by these fungal pathogens are mainly characterized by formation of round lesions on the fruit surface and deterioration in quality. Conidia are disseminated by rain splash and winds. Fungal spores germinate on aerial portions of the plants and cause anthracnose on leaves, stems, and fruits. Management of such plant diseases mainly involves the application of chemical fungicides. However, since the fungicides damage the environment and human health, alternative and more sustainable strategies are required. Although the development of new chemically synthesized fungicides is necessary, their introduction into crop systems takes a long application period, and resistant field isolates of the pathogen generally appear very quickly, within a few seasons of the introduction of a new fungicide [1,2]. Alternatively, disease management with microbial cultures or compounds derived from antagonistic microorganisms is a safer and more environmentally friendly option for consumers.

Several strains of *Bacillus subtilis* and *Bacillus amyloliquefaciens* have been reported to be effective in the biocontrol of several soil-borne plant diseases [3,4,5,6,7]. Their protective effect may depend on various mechanisms for direct antagonism of plant pathogen growth. This antagonistic activity is a consequence of antibiotic production and is well documented as an important mechanism capable of inhibiting the growth of pathogens within host plants [8,9]. In particular, the production of significant numbers of antifungal secondary metabolites, such as non-ribosomally synthesized cyclic lipopeptides (CLPs) in some *Bacillus* strains, is a major mechanism of action against pathogens [10,11]. According to their structure, these metabolites are classified into three families: surfactin, fengycin, and iturin [12]. CLPs are of variable types and comprise unusual amino acids. Furthermore, the length and composition of the fatty acid side chains provide significant structural diversity that affects the physicochemical properties and biological activities of CLPs [13]. Different homologous compounds for each CLP family are, hence, typically co-produced [14]. CLPs are crucial in the antagonism against plant pathogens and exhibit better properties than antibiotics, such as low toxicity, wide antimicrobial spectrum, and high biosurfactant activity. Iturin- and fengycin-like CLPs display strong antifungal activity and inhibit the growth of a wide range of plant pathogens [12]. These metabolites and compounds can be produced by the biocontrol agent in a formulation prior to application and have a mechanism of action based on direct antibiosis. Herein, we describe the effect of secondary metabolites produced by *B. subtilis* KB21 (Bs KB21) against phytopathogenic fungi both in vitro and *in planta*, depending on the culture medium. Their effect was determined using the following four steps: characterization of their in vitro and in vivo antifungal activity against pepper anthracnose, identification and quantification of the CLPs produced, determination of the CLPs responsible for antifungal activity, and role of CLPs in activation of defense related genes in fruit.

## 2. Results and Discussion

### 2.1. Identification of Bs KB21 Strain

**Figure 1:**
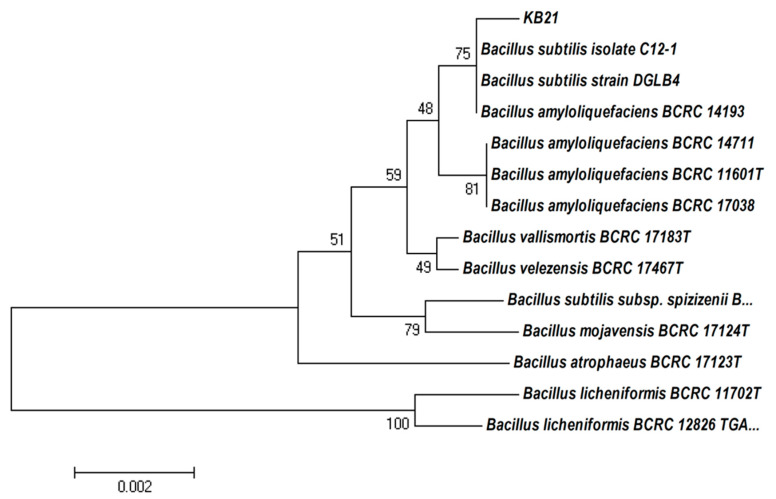
Characterization and identification of strain KB21. Neighbor-joining tree showing phylogenetic positions of strain KB21 and representatives of related taxa, based on 16S rRNA gene sequences. Bootstrap values (expressed as percentages of 1000 replications) are shown at branch points.

To determine the phylogenetic placement of strain KB21, its 16S rRNA gene sequence (1549 bp) was compared with those of some *Bacillus* species. The database of sequences was processed using the BLAST tool provided by NCBI GeneBank (http://www.ncbi.nlm.nih.gov/ (accessed on 1 April 2022)). The 16S rRNA sequences of diverse *Bacillus* species were highly similar to those of *B. subtilis* (99.7%), *Bacillus velezensis* (99.6%), and *B. amyloliquefaciens* (99.6%). These results enabled the construction of a phylogenetic tree. Phylogenetic analysis of the 16S rRNA gene sequences showed that strain KB21 and *B. subtilis* were clustered together (Figure 1). Thus, the KB21 isolate was identified as *B. subtilis* using 16S rRNA sequencing based on nucleotide homology and phylogenetic analysis. When the sequence analysis data for strain KB21 were compared with those of other *Bacillus* species, it became clear that strain KB21 is very close to phylogenetically to these *Bacillus* species.

### 2.2. Biosurfactant Production by Bs KB21 in the Cell Growth Phase

**Figure 2:**
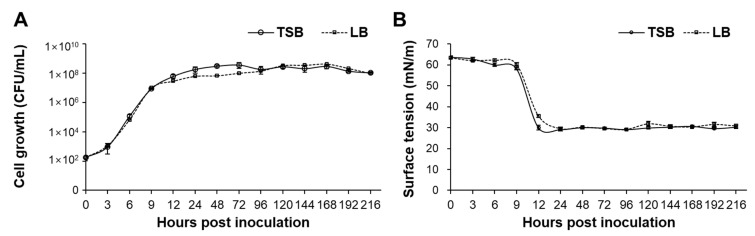
Time course profiles of bacterial growth (**A**) and production of lipopeptide biosurfactants (**B**) in *B. subtilis* KB21 fermentation. The surface tension of water control was 74.1 ± 0.1 mN/m. Data represent means ± standard errors of three replicates. TSB, Strain was cultured in Tryptic soy broth; LB Strain was cultured in Luria-Bertani.

Figure 2 depicts the pattern of cell growth and surface tension of the resulting cell culture. The cell growth was determined by determining the CFU count of the culture on TSB agar plates. Bs KB21 passed the lag phase (9.3 × 10^2^ CFU/mL) at 3 h post inoculation; the exponential phase extended to 9–12 h post inoculation (8.9 × 10^6^–6.2 × 10^7^ CFU/mL). After 24 h of growth in TSB medium, Bs KB21 entered the stationary phase (1.8 × 10^8^ CFU/mL), as indicated by the decline in the growth curve (Figure 2A). The growth of Bs KB21 in LB medium was also similar to the results of TSB culture. These results indicate that the cell growth pattern was distinct in the exponential and stationary phases. 

The surface tension of the culture filtrate for each growth phase decreased from an average of 63.7 to 30.0 mN/m and was then stable at 30.8 to 30.2 mN/m after the stationary phase (Figure 2B). The surface tension of the culture filtrate was the lowest (29.1 mN/m in TSB culture) in the stationary phase, at 24 h after inoculation. Biosurfactant production by strain KB21 occurred throughout the culture period and continued even when all of the residual nutrients were consumed. It has been reported that most of the biosurfactants produced and accumulated during the growth period between the log and stationary phases decrease the surface tension [15,16]. The maximum yield of antifungal compounds (CLP production) by *Bacillus* spp. was observed during the stationary phase. However, this yield did not correlate with the highest biosurfactant activity [17,18].

### 2.3. Antifungal Activity of Biosurfactants of Bs KB21

**Figure 3:**
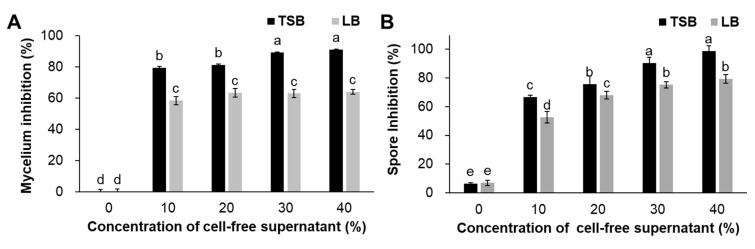
Effects of cell-free supernatants of *B*. *subtilis* KB21 on inhibition of (**A**) mycelial growth and (**B**) spore germination of *Colletotrichum* *acutatum*. The mycelium inhibition rate of the KB21 culture filtrate was calculated using the following formula: mycelia growth inhibition rate (%) = (A-B)/A × 100, where A is mycelial growth on control (0%) PDA and B is growth on PDA plates containing cell-free supernatant (10–40%). The spore germination rate calculation was as follows: spore germination rate (%) = (number of germinated spores/total number of observed spores) × 100. Data represent means ± standard errors of three replicates. The values followed by different letters in a column were significantly different at *p* < 0.05 by Duncan’s multiple range test. 10, 20, 30, 40, PDA amended with different concentrations of *B*. *substilis* KB21 culture filtrate; 0, control (PDA without Bs KB21 filtrate).

Cell-free culture filtrates derived from Bs KB21 in TSB and LB culture were tested for antifungal activity towards pepper anthracnose (Ca). The filtrates strongly affected both mycelial growth and spore germination of the plant pathogens (Figure 3). Bs KB21 filtrates caused the highest inhibition of mycelial growth of TSB, followed by LB, at the same concentrations (Figure 3A). At 10% biosurfactant content, the inhibition rate of mycelial growth was 79.4–58.3%. TSB filtrates incubated at 40% levels induced the highest reduction in Ca growth (91.1% compared to that of the untreated control). The decrease recorded in Ca growth was significantly (*p* < 0.05) different from that of both TSB and LB with filtrates. However, there were no significant (*p* < 0.05) differences in the inhibitory effects on Ca growth of TSB filtrates at 30% and 40% concentrations. In addition, different concentrations of cell-free supernatants significantly suppressed spore germination (Figure 3B). Mycelial growth was relatively less sensitive to spore inhibition, but still showed averages of 66.6% and 52.6% reductions at the 10% level for TSB and LB. The highest rate of inhibition of spore germination compared to that in the control medium was observed in TSB filtrates at 40% levels. The 30% concentration of supernatants inhibited more than 90% spore germination in TSB medium compared to the control for Ca.

**Figure 4:**
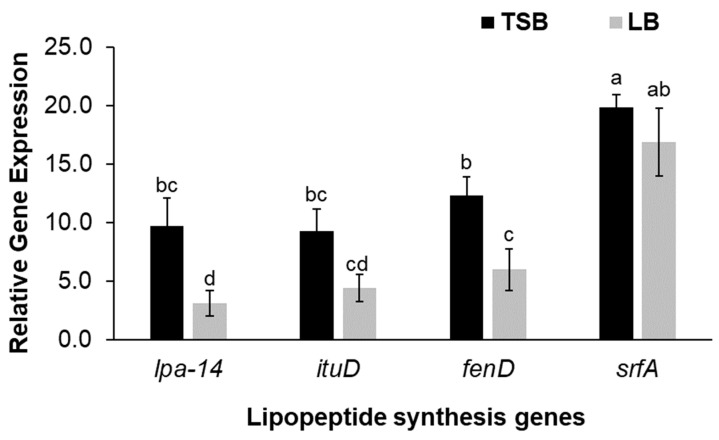
Relative expression of *lpa-14a, ituD, fenD*, and *srfA* genes after growth for five days in TSB and LB media. qRT-PCR analysis was performed using primers specific to *lpa-14, ituD, fenD* and *srfA* genes, and the expression levels were normalized against 16s rRNA gene levels. The relative mRNA levels of genes were determined by normalization of the value of Ct of the studied genes to the mean value of Ct of reference genes (16s rRNA) by means of analysis of the value 2^−∆Ct^. The values followed by different letters in a column were significantly different at *p* < 0.05 by Duncan’s multiple range test.

The cell-free supernatants of Bs KB21 effectively inhibited both mycelial growth and sporulation of plant pathogens. However, the results of antifungal activity depended on the culture nutrients. Here, we wondered whether there was a difference in antifungal activity depending on the culture source, so we decided to find the produced antifungal active material. The CLP families produced by the Bs KB21 were analyzed in each medium. To identify the mechanism underlying these phenomena (Figure 3), we assessed the transcriptional level of CLP synthesis genes (*lpa-14. ituD, fenD, srf A*) expression at 5 days. The relative expression levels of CLP synthesis genes were upregulated in the TSB culture compared to LB culture (Figure 4). The mRNA levels of iturin A and fengycin synthesis-related gens were higher in Bs KB21 grown in TSB than in the LB culture. The expression levels of the *lpa-14* and *fenD* were 6.6 and 6.3-fold higher in the TSB culture, respectively, than in the LB culture.

Biosurfactants affect the proportion of CLP family compounds and the formation of specific CLP types, resulting in selective antifungal effects. Biosurfactants produced by *Bacillus* strains have been reported to exhibit antifungal activity against phytopathogenic fungal diseases, and the most commonly reported class of biosurfactants with antifungal activity is CLPs [12]. These *Bacillus* lipopeptides are hypothesized to be important in the biological control potential of the strain. The lipopeptide antibiotics produced by *B. subtilis* identified to date are surfactin, iturin, fengycin, mycosubtilin, and bacillomycin. The production of CLPs closely correlates with nutrient substrates such as carbohydrates, vegetable oil, starch, and hydrocarbons [19,20]. Since the synthesis and yield of CLP components may be influenced by the composition of the medium and fermentation conditions [21,22], it is necessary to select nutrient substrates for the maximum yield of specific CLPs. Therefore, Bs KB21 can be used as a more effective biological agent for controlling the growth of phytopathogenic fungi by optimizing the fermentation conditions for CLP production and enabling the generation of stable and effective CLPs.

### 2.4. Identification of CLPs from Bs KB21 Culture Filtrate

**Figure 5:**
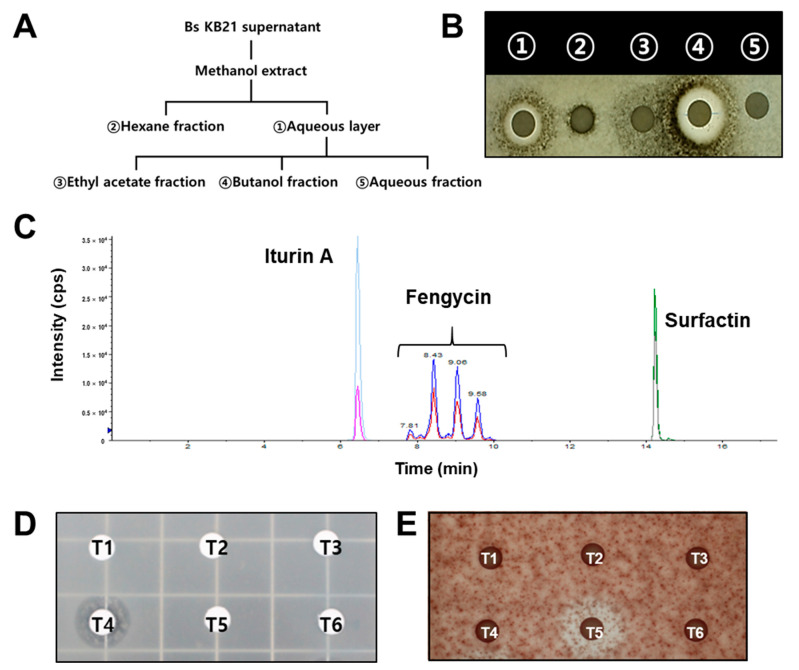
Characterization and identification of lipopeptides (CLPs) in butanol extracts of *B*. *subtilis* KB21. (**A**) Procedure for extraction of compounds from the cell-free supernatants. (**B**) Antifungal activity of crude extracts isolated from *B*. *subtilis* KB21. 1, Compounds extracted from the aqueous layer in Figure 5A; 2, Compounds extracted from the hexane solvent; 3, Compounds extracted from the ethyl acetate; 4, Compounds extracted from the butanol; 5, final aqueous solution. (**C**) Peaks of purified fractions analyzed by LC-MS/MS. (**D**) Inhibitory activity of purified fractions CLPs from butanolic extracts on *C*. *acutatum* spore growth. (**E**) Inhibitory activity of fractions CLPs on *Fusarium oxysporum* spore growth. T1, T2, T3, T4, T5, purified fractions CLPs from *B*. *substilis* KB21; T6, methanol control.

The antifungal activity of crude extracts against spore germination of anthracnose pathogens was assayed to identify the active compounds in the extracts (Figure 5). When the crude extracts were applied at 30 μg/mL for controlling pepper anthracnose, the butanol and aqueous layer fractions more strongly inhibited spore germination compared with that by other treatments (Figure 5B). The crude extracts with antifungal activity were analyzed using TLC and LC-MS. The crude extracts of Bs KB21 in TSB supernatants were separated initially on TLC plates. These plates showed spots with R*_f_* values similar to those of standard fengycin (R*_f_* = 0.09–0.2), iturin A (R*_f_* = 0.3–0.5), and surfactin (R*_f_* ≥ 0.7). The products were found to contain cyclic peptides and were regarded as putative CLPs. The homologous CLPs of the three families produced by Bs KB21 in TSB medium were analyzed by LC-MS (Figure 5C). The retention times, total ion chromatograms, and peak areas on the chromatograms obtained from the samples were compared with those of the standards for identification and quantification. The products were identified as CLPs with molecular ion peaks [M + H]^+^ and mass spectra identical to those of iturin A, surfactin, and fengycin standards. The results showed that the appearance of the product ions was regular. Product ion fragments were identified from the MS/MS spectrum with the molecular ion peaks [M + H]^+^ *m/z* 1008, 1022, 1036, 1044, and 1058, coinciding with the predicted mass of surfactin (Figure S1). They corresponded to the known standard of surfactin with fatty acid side chains. Iturin A showed four primary fragile single peaks at *m/z* 1043, 1057, 1065, and 1079. The peak at *m/z* 1043 was similar to that of the lipopeptide, iturin (Figure S2), and the isoform peaks at *m/z* 1057, 1065, and 1079 were deduced according to the number of carbon atoms of their fatty acid side chains. The ESI-MS spectra of putative fengycin purified from the culture of strain KB21 revealed several molecules that were observed at [M + H]^+^ *m/z* 1447, 1464, 1475, 1477, 1491, and 1505 (Figure S3).

### 2.5. Identification and Analysis of Antifungal CLPs from Bs KB21

The further fractionation of the biosurfactants using butanol as solvent was analyzed by TLC. The isolated fractions (T1–T5) appeared as several spots on the TLC plate, and the CLPs were identified using MS (Table 1). The fractions from the butanol extract produced spots corresponding to fengycin, iturin A, and surfactin. The mycelial growth inhibition assay was performed to identify the antifungal CLPs among fractions T1–T5. Only the T4 fraction strongly inhibited the radial growth of Ca; the other fractions of the butanol extract did not effectively inhibit fungal spore growth (Figure 5D). However, the T5 fraction strongly inhibited spore growth against the Fo pathogen but not the anthracnose pathogen (Figure 5E). CLP quantification indicated that the T4 fraction contained the highest levels of iturin (69.2 mg/kg), whereas the other fractions predominantly contained surfactin and fengycin (Table 1). These results showed that the antifungal activity of iturin-like CLPs was directly related to the suppression of the mycelial growth of anthracnose pathogens. Moreover, the T4 fraction sprayed on pepper fruits showed similar antifungal activity. The disease severity (11.9%) in fruits treated with 60 μg/mL T4 decreased by 73% compared with that in pepper fruits treated with T6 control (84.9%). This indicated that iturin-like CLPs (T4 fraction) exerted an antifungal effect by reducing pepper anthracnose symptoms.

*Bacillus* spp. are known to use multiple antifungal compounds to suppress a specific fungal pathogen. The main CLP of *Bacillus* spp. that is known to have antifungal activity is iturin A, whereas the CLP with the highest antimicrobial activity is fengycin [12,17,23]. Therefore, it appears that different sets of CLPs may be involved in combatting fungal pathogens and in generating a strain-specific response. Notably, the application of these CLPs resulted in the disruption of the penetration ability of fungal pathogens and in the progression of infection in pepper plants. Reports have shown that the cell membrane of fungal pathogens is the site of antifungal attack by CLPs, and that iturin CLPs exert their antifungal activity by altering the integrity of the fungal cell wall and modulating membrane permeability [24,25]. In biological membrane models, iturin can also alter the target cell membranes and the cell membrane structure in a dose-dependent manner [26,27]. Additionally, it can pass through the cell wall, damage the cell membrane, and cause changes in membrane permeability, leading to the loss of K+ ions, thereby inhibiting the fungal infection [28,29]. Therefore, the production of iturin-like CLPs may be related to the production of biocontrol agents by vegetative cells in response to pepper anthracnose. These findings suggest that the application of CLPs is a more effective strategy than chemical treatment for reducing the incidence and severity of pepper anthracnose.

### 2.6. Antifungal Activity of Iturin-Like CLPs Isolated from Bs KB21 against Ca

**Figure 6:**
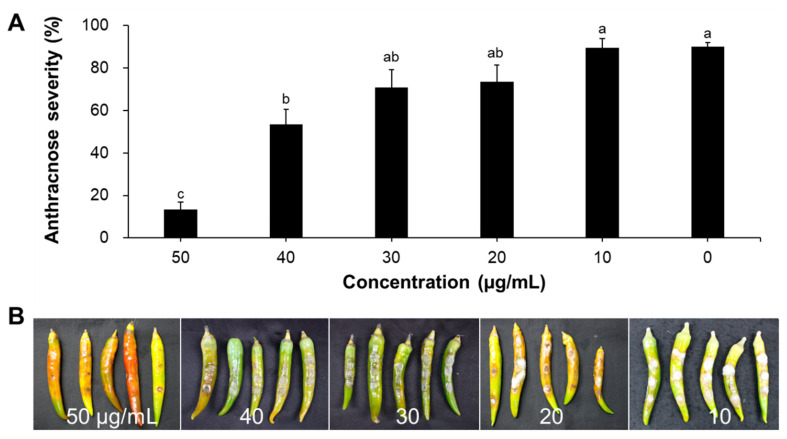
Antifungal activity of iturinic lipopeptides isolated from *B*. *subtilis* KB21. (**A**) Disease severity of pepper anthracnose upon treatment with fractioned lipopeptides (10, 20, 30, 40, and 50 μg/mL). (**B**) Photo taken seven days post-inoculation. Data are means ± standard errors of three replicates. The values followed by different letters in a column were significantly different at *p* < 0.05 by Duncan’s multiple range test.

To confirm whether the antifungal activity of the T4 fraction of TSB culture on pepper fruits can be attributed to the iturin-like CLPs produced by strain KB21, CLPs from butanol fractions were applied to pepper fruits (Figure 6). The iturin fractions provided 18.5–85.2% greater control efficiency than that by water treatment. In addition, various concentrations (50, 40, 30, 20, and 10 μg/mL) of iturin fractions reduced the anthracnose symptoms on pepper fruits compared to those on pathogen-inoculated controls. When the 50 μg/mL iturin fraction was used to treat pepper anthracnose, the disease incidence in pepper fruits reduced by 76.7% compared with that in the controls. Other treatments also showed similar antifungal activity compared to the control, but the results at the 10 μg/mL level were not significant. The EC_50_ value of the iturin fractions for efficient control of pepper anthracnose on fruits was 41.7 μg/mL. Consistent with the crude extract treatments, the disease severity (formation of lesions) was significantly lower depending on the dosage. Correspondingly, the extracts suppressed disease progression at significantly lower concentrations compared to that in the water treatment control. The symptoms of the disease in fruits initially began as water-soaked lesions that became soft and slightly sunken and tan in color. The T4 fraction showed the highest activity against pepper anthracnose, as demonstrated by its inhibition of fungal growth and disease incidence both in vitro and in vivo. In addition, it showed the highest iturin A content, based on the results of LC-MS analysis. The application of iturin A lipopeptide to pepper fruits resulted in the lowest disease incidence in the fruits compared to that in the untreated control (*p* < 0.05). However, the T4 fraction showed different results against tomato Fusarium wilt and pepper *Cucumber mosaic virus* (data not shown). Moreover, there was no correlation between the total content of CLPs and the antifungal activity. The disease control activity of iturin-like CLPs could be attributed to the presence of biologically active ingredients, which have direct antifungal activity and can reduce the development of pepper anthracnose. Therefore, specific CLPs appear to be more effective biocontrol agents based on plant-pathogen interactions. 

### 2.7. Induction by Iturin CLP on the Defense Related Gene Transcript in Pepper Anthracnose

**Figure 7:**
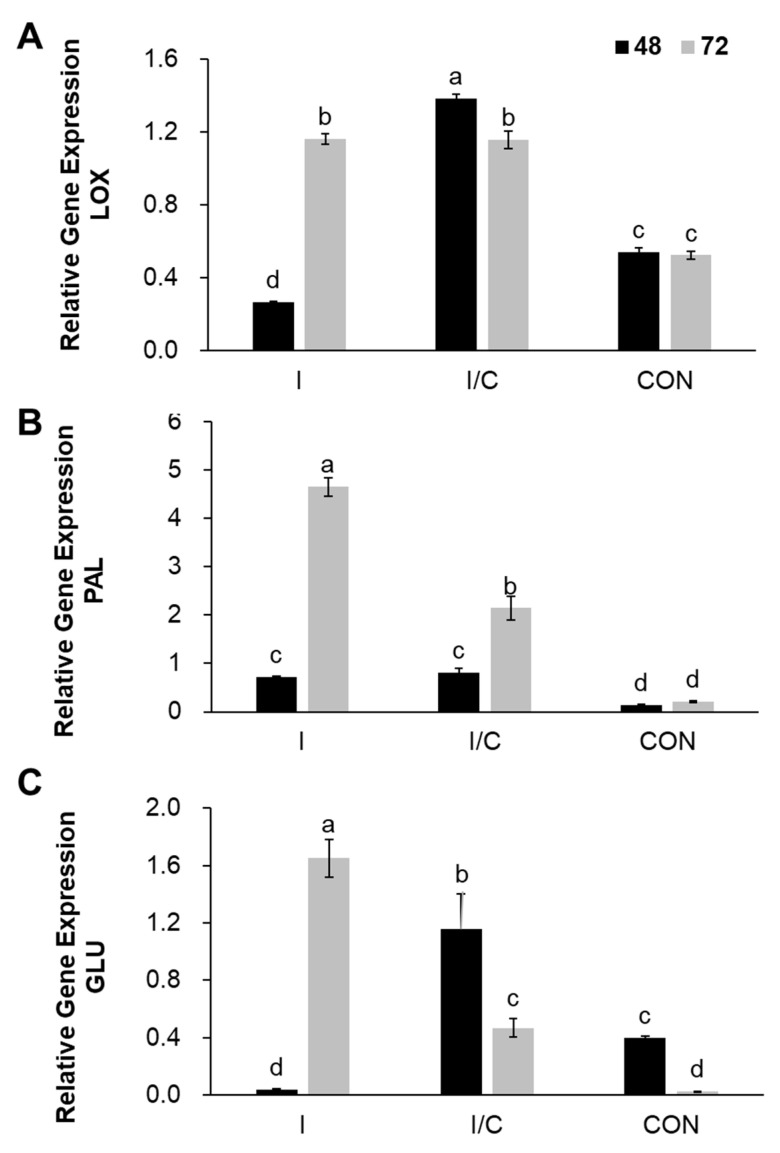
qRT-PCR for defense-related genes (*LOX*(**A**) *PAL* (**B**), and *GLU* (**C**)) in pepper fruits treated with purified iturin CLP isolated from Bs KB21 culture. Samples were collected at 48 and 72 h post-Ca inoculation. The relative expression of defense-related genes was monitored using qRT-PCR at 48 h and 72 h after anthracnose pathogen (Ca) inoculation. The housekeeping gene *C. annuum* actin was used for normalization. The relative expression levels represent the means of three independent experiments, and different letters indicate significant differences (*p* < 0.05) in transcript expression against Ca according to Duncan’s multiple range tests. Error bars represent means ± standard error. I, Application of 66 μg/mL iturin CLP (in 0.1% DMSO) isolated from Bs KB21; I/C, Application of iturin CLP isolated from Bs KB21 24 h prior to inoculation with Ca; CON, Application of 0.1% DMSO 24 h prior to inoculation with Ca (control). LOX; Lipoxygenase, PAL; Phenylalanine ammonia lyase, and GLU; β-1,3-glucanase.

To confirm the effects of iturin CLP on induction of defense-related genes, JA (*LOX*), SA (*PAL*) signaling pathways and on transcription pattern of *GLU* in pepper fruits treated with pathogen attack was conducted. The relative expression of defense-related genes was monitored using qRT-PCR at 48 h and 72 h after anthracnose pathogen inoculation (Figure 7). The expression of all genes tested in fruits treated with iturin CLP, iturin CLP + Ca (application of 66 μg/mL iturin CLP isolated from Bs KB21 24h prior to inoculation with Ca) was upregulated to varying degrees when compared with that in the control fruits. The pepper fruits of iturin CLP without pathogens treatment increase rapidly at 72 h. In addition, *LOX, PAL*, and *GLU* transcription levels in iturin CLP treated group were 0.6-fold, 4.4-fold, and 1.6-fold higher, respectively, than those in the control at 72 h (*p* < 0.05). However, in iturin CLP + Ca treatment group, only *PAL* transcription level increased 2.1-fold at 72 h, and the expression level of other genes decreased. These results demonstrate that iturin CLP-produced Bs KB21 fermentation stimulates various defense responses in the pepper plant. Previous studies have shown that host plants inoculated with biocontrol microorganisms increase the expression of defense-related genes in plant diseases. 

In this study, we have confirmed that iturin CLP produced by the antagonistic Bs KB21 elicit the induction and expression of defense-related genes in pepper fruit. in addition, treatment with iturin CLP isolated from Bs KB21 enhanced plant defense responses through modulation of SA- and JA-dependent pathways in generating signal molecules for activating ISR mechanism. Furthermore, iturin CLP stimulated the gene *GLU*, which has a direct effect on the pathogens. This study showed a strong gene expression of *GLU* in fruit induced by both iturin CLP and the pathogen. *GLU* and *LOX* expression were obviously shown higher levels in iturin CLP + pathogen treated fruits than when treated with the pathogen alone. Iturin CLP from Bs KB 21 fermentation exerted a protective effect on plants through stimulation of plant immune system. *Bacillus* CLPs have similar overall structures, but their biological activities vary. Iturin and fengycin exhibit potent antifungal activity, so they may play a dual or selective role in reducing plant diseases. Therefore, the reduction in fungal severity associated with iturin CLP treatment can be attributed to the direct antifungal effect and induction resistance due to its synergistic effect with fengycin.

## 3. Materials and Methods

### 3.1. Identification of Bs KB21 and Fungal Pathogens

Bs KB21 used in this study was isolated from a rice paddy field; soil from the field was diluted with sterile water, spread on tributyrin agar (KisanBio Co., Ltd., Seoul, Korea), and cultured at 28 °C for three days. Cells of the isolated strain were added to 20% glycerol, stored at −80 °C, and used for identification and activity assays. 16S rRNA gene analysis was performed for biological identification of the isolated strain using the genetic sequence characteristics. The strain was cultured in tryptic soy broth (TSB; Becton Dickinson GmbH, Heidelberg, Germany) or Luria-Bertani (LB; Becton Dickinson GmbH), and a genomic DNA Prep kit (NanoHelix Co., Ltd., Daejeon, Korea) was used to extract genomic DNA. The universal primers, 27F (5′-AGAGTTTGATCATGGCTCAG-3′) and 1492R (5′-GGATACCTTGTTACGACTT-3′), were used for 16S rRNA gene amplification. Nucleotide sequence analysis was performed by Solgent ASSA (Solgent Co., Ltd., Daejeon, Korea). Sequence data were compared with available data by BLAST analysis using the NCBI sequence databank. Relevant sequences were collected, data were plotted with Mega 7.0 software, and a phylogenetic tree was constructed. The isolate, Bs KB21, was grown in solid or liquid TSB. Cultures were incubated with shaking (120 rpm) at 28 °C. Vegetative cell growth was measured as optical density at 600 nm, and colony-forming units (CFUs) were determined by plating 10-fold serial dilutions onto TSB agar plates.

The phytopathogenic fungi, *Fusarium oxysporum* KACC 40046 (Fo) and *Colletotrichum* *acutatum* KACC 40689 (Ca), were obtained from the Korean Agricultural Culture Collection (National Agrobiodiversity Center, Suwon, Korea). They were incubated on potato dextrose agar (PDA; Becton Dickinson GmbH) at 25 °C for seven days. Under sterile conditions, fungal culture plates were flooded with sterile distilled water, and fungal suspensions were filtered through three layers of sterile cheesecloth to remove mycelia. The conidial concentration was determined using a hemacytometer (Marienfeld Superior, Lauda-Königshofen, Germany) and adjusted to 1 × 10^5^ conidia/mL.

### 3.2. Fungal Growth Inhibition Assay

To evaluate the production of antifungal compounds, strain KB21 was cultured with shaking at 150 rpm in a TSB medium for five days. Bacterial cells were pelleted from the culture broth by centrifugation for 30 min at 8000× *g* and 4 °C. The supernatant was filtered through 0.2 μm cellulose nitrate filters (Millipore Filter Corp., Bedford, MA, USA) to remove all cells. Cell-free filtrates were diluted with the PDA medium to different concentrations (0%, 10%, 20%, 30%, and 40%) of the cell-free culture supernatant of strain KB21 grown to stationary phase. They were poured into Petri dishes (9 cm, SPL Life Sciences Co., Pocheon, Korea). Mycelial growth inhibition was tested by placing 5 mm agar discs containing plant fungal pathogens (taken from five-day plates) at the center of PDA plates containing cell-free supernatants of TSB cultures. The diameter of mycelial growth was measured after seven days at 28 °C by image analysis using APS assess 2.0 imaging software (APS, St. Paul, MN, USA). The percentage mycelium inhibition rate of the KB21 culture filtrate was calculated using the formula, mycelial growth inhibition (%) = (A − B)/A × 100, where A is mycelial growth in the control PDA and B is mycelial growth in the amended PDA plates.

The inhibition assay of fungal spore germination was performed in a sterile culture plate (24 wells; SPL Life Sciences Co.). To determine the effects of the cell-free supernatant and CLPs, their inhibitory activity against anthracnose pathogens was examined. Sterilized water or methanol solution (negative control) and PDB (positive control) were included in the germination experiments as controls. The cell-free supernatant and extracted CLPs were mixed with spore suspensions (1 × 10^4^ conidia/mL) and incubated at 28 °C in the dark while shaking at 200 rpm. Spore germination was determined microscopically after 24 h by counting 200 conidia/well. A conidium was considered germinated if the germ tube length was at least equal to the spore length. The experiment was performed in triplicate. 

### 3.3. Antifungal Activity of Clps from Bs KB21 on Pepper Fruit

Pepper anthracnose assay was performed using the fruit puncture method. Three sets of five postharvest fruits (cv. Chengyang) each at the matured green ripened stage were used. Ten microliters of the conidial suspension (10^5^ CFU/mL) was inoculated into the wounds in the fruits using a sterile cork-borer of 5 mm diameter before the extracts were applied on the fruit. The treatment agents for the in vivo assay were CLP extracts and controls. Injected fruits were incubated in a plastic moisture chamber at high relative humidity (~95–98%) at 28 °C for 24 h. The disease severity was determined based on the mean percentage lesion size, which was converted to disease severity scores of 0–5. Disease severity was assessed as sunken circular depression of the fruit surface as follows: 0, no lesion; 1, 1–2% of the fruit area shows necrotic lesion or a larger water-soaked lesion surrounding the infection site; 2, >2–5% of the fruit area shows necrotic lesion and acervuli or a water-soaked lesion up to 5% of the fruit surface; 3, >5–15% of the fruit area shows necrotic lesion and acervuli or water-soaked lesion up to 25% of the fruit surface; 4, >15–25% of the fruit area shows necrotic lesion with acervuli; 5, >25% of the fruit area shows necrosis, lesion often encircling the fruit, and abundant acervuli. Based on infection counts and disease symptom assessments, the disease severity index was calculated as follows: Disease severity (%) = (∑(rating × number of fruits rated)/total number of fruits × highest rating) × 100. The experiment was repeated with three replicates of five fruits per treatment.

### 3.4. Isolation and Identification of Antifungal Clps

Biosurfactants produced by Bs KB21 were collected from TSB culture for 7 days post inoculation. Bacterial cells were pelleted from each culture broth by centrifugation for 30 min at 8000× *g* at 4 °C, and the supernatants were filtered through 0.22 µm filters (Millipore Filter Corp.) to ensure cell removal. To estimate biosurfactants production (*Bacillus* lipopeptides), surface tension (mN/m) of the cell-free supernatants was measured using the Ring method [30] with a surface tensiometer (K6; KRÜSS GmbH, Hamburg, Germany) at 25 °C with three replicates/sample. The average value was used to express the surface activity of each sample. To calibrate the instrument, the surface tension of pure water control was measured.

The secondary metabolites produced in the culture medium of the strain were extracted using methanol to analyze the antifungal active compounds. The crude extracts were further extracted with n-hexane, a non-polar solvent, and the aqueous layer was sequentially extracted three times using ethyl acetate and butanol. The solvent from the extracted compounds was evaporated under vacuum before dissolving the compound in methanol and analysis by TLC (silica gel 60; Merck, Darmstadt, Germany) using chloroform:methanol:water (65:25:4, *v*/*v*/*v*) as the mobile phase [31]. The butanol extract was separated by preparative TLC (Prep TLC; 20 × 20 cm, 0.5 mm, Merck), and five regions were scraped from the prep TLC plate and then fractioned with methanol.

### 3.5. Quantitative Analysis of Antifungal Clps Using Liquid Chromatography-Tandem Mass Spectrometry (LC-MS/MS)

Quantitative analyses were performed using LC-MS/MS (ABSCIEX mass spectrometer, ABSCIEX, MA, USA) with a Capcell Core C_18_ HPLC Column (2.1 × 150 mm, 2.7 um; OSAKA SODA, OSAKA, JAPAN) at a flow rate of 0.3 mL/min. Samples were run with a flow rate of 10 μL/min at 40 °C in a positive mode. To detect iturin, surfactin, and fengycin simultaneously, a binary solvent system was run in gradient mode with mobile phase A (0.1% formic acid in water) and mobile phase B (0.1% formic acid in methanol) in the following sequence: 15% B (0–1 min), increased to 60% (1–1.5 min) and then 90% (1.5–10 min) and maintained at 90% B (10–12 min). Then, the gradient was set to 98% B (12–12.1 min), maintained at 98% B (12.1–16 min), after which the column was set to 15% B (16–16.1 min) and equilibrated (16.1–25 min). All compounds were ionized in electro spray ionization (ESI) mode and analyzed in multiple reaction monitoring (MRM) mode (Table S1). The precursor ion, product ions, and collision energy were optimized via direct injection of the individual pesticide standard solution (0.1 μg/mL) into the mass spectrometer. Analyst (Version 1.6, ABSCIEX) and Multiquant (Version 3.0.2, ABSCIEX) software were used for instrument control, data acquisition, and processing. The compounds were identified and quantified by comparing their retention times and masses with those of commercial standards, as described by Kang et al. (2020) [17]. Fengycin, surfactin, and iturin A standards were purchased from Sigma-Aldrich (St. Louis, MO, USA). The curves of peak height for known concentration were created with four calibration points for each surfactant. The areas of the quantified ions were used for calculations.

### 3.6. Quantification of Lipopeptide Biosurfactants and Defense-Related Gene Expression Using qPCR- RT

Quantification analyses were performed with relative gene expression using Bio-Rad CFX connect Real-Time PCR Detection System (Bio-Rad, CA, USA) and specific primers. Total RNA was extracted from Bs KB21 grown in culture media or the fine powder of the treated pepper tissue using TRIzol Reagent (Invitrogen, Carlsbad, CA, USA), as per the manufacturer’s instructions. The relative expression of target gene was quantified using the 2^−ΔΔCT^ method [32]. PCR was performed in a reaction mixture comprising iScript cDNA synthesis kit (Bio-Rad Laboratories), SuperScript One-step RT-PCR (for lipopeptide gene expression, Invitrogen), iQ Sybr Green supermix kit (Bio-Rad Laboratories), and 10 pM of each primer (Table 1.). The primers used for qRT-PCR analysis of lipopeptide biosurfactants and defense-related genes were as follows: Iturin synthesis gene, *lpa-14* (forward 5′-TCATCTCAATCCCGTCACAA-3′ and reverse 5′-TATCAAACAGGCCGGAAAAG-3′), and *ituD* forward 5′-CTCAAGCAGCACATGACGAT-3′ and reverse 5′-ACCGGCTAAGACATTGTTCG-3′); Fengycin synthesis (*fenD*), forward 5′-GGCCCGTTCTCTAAATCCAT-3′ and reverse 5′-GTCATGCTGACGAGAGCAAA-3′’; Surfactin synthesis (*srfA*), forward 5′-TCGGGACAGGAAGACATCAT-3′ and reverse 5′-CCACTCAAACGGATAATCCTGA-3′; β-1,3-glucanase (*GLU*), forward 5′-CGTTACAGCTGCCCTATCTCAA-3′ and reverse 5′-GTTTGTTGTCGTGGCAAATGC-3′; Lipoxygenase (*LOX*), forward 5′-TGGTGATCCTGCGAATGGTT-3′ and reverse 5′-CGTCCCAATCAAACGTGACA-3′; Phenylalanine ammonia lyase (*PAL*), forward 5′-AAGTCATTCGCGCTGCAACT-3′ and reverse 5′-CCACCGTGTAAGGCCTTGTT-3′; *C. annuum* actin, forward 5′-CACTGAAGCACCCTTGAACCC-3′ and reverse 5′-GAGACAACACCGCCTGAATAGC-3′. Three replicates were used for data analysis.

### 3.7. Statistical Analysis

All experiments were performed at least three times per treatment. Data are expressed as means ± standard errors. The data were analyzed using one-way analysis of variance, followed by Duncan’s multiple range tests (*p* < 0.05 was considered significant). All statistical analyses were performed using SPSS v23.0 (SPSS Inc., Chicago, IL, USA).

## 4. Conclusions

In this study, the CLPs produced by Bs KB21 were proven to be competent biocontrol agents for plant fungal pathogens. Comprehensive chemical analysis of KB21 culture filtrates confirmed the presence of three different lipopeptide antibiotics, surfactin, fengycin, and iturin A, compounds well known for their strong antifungal effect against different phytopathogenic fungi. In addition, the *m*/*z* of their peaks showed high similarity to the CLP homologues of the standards. CLP groups confer an advantage to the *Bacillus* strains producing them in specific ecological niches. Overall, the results of this study demonstrate that the control of pepper anthracnose by strain KB21 most probably relies on such lipopeptide-dependent direct antagonism of the bacteria toward fungal development. The growth of strain KB21 in the TSB medium resulted in enhanced biomass and CLP yields. Our results also suggest that the application of CLPs produced by strain KB21 is an efficient alternative for controlling the pathogenesis of Ca in pepper plants. We demonstrated that the high antifungal activity and induced resistance of CLPs is due to the production of iturin. This study provided new evidence for the poorly characterized role of the iturin-type fraction produced by *Bacillus* spp. as an antifungal compound and highlighted the possibility of using CLPs for controlling pepper anthracnose caused by Ca. 

## Figures and Tables

**Table 1:** Quantification and antifungal activity of fractions extracted from *B. subtilis* KB21.

Fraction	Lipopeptide Quantification (mg/kg)			Antifungal Activity	
	Iturin	Surfactin	Fengycin	Mycelial Growth	Pepper Fruit (%)
T1	0.1 ± 0.06	11.1 ± 6.74	0.9 ± 0.52	-	77.6 ^b^
T2	0.0 ± 0.03	0.6 ± 0.39	0.9 ± 0.55	-	84.2 ^a^
T3	0.1 ± 0.04	600.0 ± 18.23	2.9 ± 1.77	-	71.7 ^bc^
T4	69.2 ± 3.97	2.8 ± 0.71	1.3 ± 0.65	+	11.9 ^d^
T5	0.5 ± 0.30	1.3 ± 0.80	78.1 ± 3.12	-	69.2 ^c^
T6	N/A	N/A	N/A	-	84.9 ^a^

Values represent the means of peak heights for standards measured by LC-MS/MS. T1–T5, Fractions isolated from strain KB21; T6, water control; N/A; not available. Data are means ± SE of three replicates. The values followed by different letters in a column were significantly different at *p* < 0.05 by Duncan’s multiple range test.

## Data Availability

Not applicable.

## Supplementary Materials

The following are available online at https://www.mdpi.com/article/10.3390/plants11091267/s1, Figure S1: Molecular ion peaks and mass spectra of surfactin of Bs KB21, Figure S2: Molecular ion peaks and mass spectra of iturin of Bs KB21, Figure S3: Molecular ion peaks and mass spectra of fengycin of Bs KB21, Table S1: MRM transition parameters of analytes in LC-MS/MS.

## References

[1] Lucas J.A., Hawkins N.J., Fraaije B.A. (2015). The evolution of fungicide resistance. Adv. Appl. Microbiol..

[2] Beckerman J.L., Sundin G.W., Rosenberger D.A. (2015). Do some IPM concepts contribute to the development of fungicide resistance? Lessons learned from the apple scab pathosystem in the United States. Pest Manag. Sci..

[3] Borriss R. (2011). Use of plant-associated *Bacillus* strains as biofertilizers and biocontrol agents in agriculture. Bacteria in Agrobiology: Plant Growth Responses.

[4] Pérez-García A., Romero D., De Vicente A. (2011). Plant protection and growth stimulation by microorganisms: Biotechnological applications of *Bacilli* in agriculture. Curr. Opin. Biotechnol..

[5] Liu D., Li K., Hu J., Wang W., Liu X., Gao Z. (2019). Biocontrol and action mechanism of *Bacillus amyloliquefaciens* and *Bacillus subtilis* in soybean phytophthora blight. Int. J. Mol. Sci..

[6] Kang B.R., Song Y.-S., Jung W.-J. (2021). Differential expression of bio-active metabolites produced by chitosan polymers-based *Bacillus amyloliquefaciens* fermentation. Carbohydr. Polym..

[7] Yan F., Li C., Ye X., Lian Y., Wu Y., Wang X. (2020). Antifungal activity of lipopeptides from *Bacillus amyloliquefaciens* MG3 against *Colletotrichum gloeosporioides* in loquat fruits. Biol. Control.

[8] Cawoy H., Bettiol W., Fickers P., Ongena M., Stoytcheva M. (2011). *Bacillus*-based biological control of plant diseases-pesticides use and management. Pesticides in the Modern World-Pesticides Use and Management.

[9] Shoda M. (2019). Biocontrol of Plant Diseases by Bacillus Subtilis: Basic and Practical Applications.

[10] Torres M.J., Brandan C.P., Sabaté D.C., Petroselli G., Erra-Balsells R., Audisio M.C. (2017). Biological activity of the lipopeptide-producing *Bacillus amyloliquefaciens* PGPBacCA1 on common bean *Phaseolus vulgaris* L. pathogens. Biol. Control.

[11] Veras F.F., Correa A.P.F., Welke J.E., Brandelli A. (2016). Inhibition of mycotoxin-producing fungi by *Bacillus* strains isolated from fish intestines. Int. J. Food Microbiol..

[12] Ongena M., Jacques P. (2008). *Bacillus* lipopeptides: Versatile weapons for plant disease biocontrol. Trends Microbiol..

[13] Dunlap C.A., Schisler D.A., Price N.P., Vaughn S.F. (2011). Cyclic lipopeptide profile of three *Bacillus subtilis* strains; antagonists of Fusarium head blight. J. Microbiol..

[14] Akpa E., Jacques P., Wathelet B., Paquot M., Fuchs R., Budzikiewicz H., Thonart P. (2001). Influence of culture conditions on lipopeptide production by *Bacillus subtilis*. Appl. Biochem. Biotechnol..

[15] Lin L.-Z., Zheng Q.-W., Wei T., Zhang Z.-Q., Zhao C.-F., Zhong H., Xu Q.-Y., Lin J.-F., Guo L.-Q. (2020). Isolation and characterization of fengycins produced by *Bacillus amyloliquefaciens* JFL21 and its broad-spectrum antimicrobial potential against multidrug-resistant foodborne pathogens. Front. Microbiol..

[16] Pathak K.V., Keharia H. (2014). Application of extracellular lipopeptide biosurfactant produced by endophytic *Bacillus subtilis* K1 isolated from aerial roots of banyan (*Ficus benghalensis*) in microbially enhanced oil recovery (MEOR). 3 Biotech.

[17] Alvarez F., Castro M., Príncipe A., Borioli G., Fischer S., Mori G., Jofré E. (2012). The plant-associated *Bacillus amyloliquefaciens* strains MEP218 and ARP23 capable of producing the cyclic lipopeptides iturin or surfactin and fengycin are effective in biocontrol of sclerotinia stem rot disease. J. Appl. Microbiol..

[18] Kim Y.T., Kim S.E., Lee W.J., Fumei Z., Cho M.S., Moon J.S., Oh H.-W., Park H.-Y., Kim S.U. (2020). Isolation and characterization of a high iturin yielding *Bacillus velezensis* UV mutant with improved antifungal activity. PLoS ONE.

[19] Ghribi D., Ellouze-Chaabouni S. (2011). Enhancement of *Bacillus subtilis* lipopeptide biosurfactants production through optimization of medium composition and adequate control of aeration. Biotechnol. Res. Int..

[20] Kang B.R., Park J.S., Jung W.-J. (2021). Antiviral activity by lecithin-induced fengycin lipopeptides as a potent key substrate against *Cucumber mosaic virus*. Microb. Pathog..

[21] Medeot D.B., Bertorello-Cuenca M., Liaudat J.P., Alvarez F., Flores-Cáceres M.L., Jofré E. (2017). Improvement of biomass and cyclic lipopeptides production in *Bacillus amyloliquefaciens* MEP218 by modifying carbon and nitrogen sources and ratios of the culture media. Biol. Control.

[22] Sun D., Liao J., Sun L., Wang Y., Liu Y., Deng Q., Zhang N., Xu D., Fang Z., Wang W. (2019). Effect of media and fermentation conditions on surfactin and iturin homologues produced by *Bacillus natto* NT-6: LC–MS analysis. AMB Express.

[23] Kang B.R., Park J.S., Jung W.-J. (2020). Antifungal evaluation of fengycin isoforms isolated from *Bacillus amyloliquefaciens* PPL against *Fusarium oxysporum* f. sp. lycopersici. Microb. Pathog..

[24] Deleu M., Paquot M., Nylander T. (2005). Fengycin interaction with lipid monolayers at the air–aqueous interface—Implications for the effect of fengycin on biological membranes. J. Colloid Interface Sci..

[25] Kim K., Lee Y., Ha A., Kim J.-I., Park A.R., Yu N.H., Son H., Choi G.J., Park H.W., Lee C.W. (2017). Chemosensitization of *Fusarium graminearum* to chemical fungicides using cyclic lipopeptides produced by *Bacillus amyloliquefaciens* strain JCK-12. Front. Plant Sci..

[26] Fiedler S., Heerklotz H. (2015). Vesicle leakage reflects the target selectivity of antimicrobial lipopeptides from *Bacillus subtilis*. Biophys. J..

[27] Zakharova A.A., Efimova S.S., Malev V.V., Ostroumova O.S. (2019). Fengycin induces ion channels in lipid bilayers mimicking target fungal cell membranes. Sci. Rep..

[28] Li T., Li L., Du F., Sun L., Shi J., Long M., Chen Z. (2021). Activity and mechanism of action of antifungal peptides from microorganisms: A review. Molecules.

[29] Thimon L., Peypoux F., Wallach J., Michel G. (1995). Effect of the lipopeptide antibiotic, iturin A, on morphology and membrane ultrastructure of yeast cells. FEMS Microbiol. Lett..

[30] Chopineau J., McCafferty F.D., Therisod M., Klibanov A.M. (1988). Production of biosurfactants from sugar alcohols and vegetable oils catalyzed by lipases in a nonaqueous medium. Biotechnol. Bioeng..

[31] Arrebola E., Jacobs R., Korsten L. (2010). Iturin A is the principal inhibitor in the biocontrol activity of *Bacillus amyloliquefaciens* PPCB004 against postharvest fungal pathogens. J. Appl. Microbiol..

[32] Livak K.J., Schmittgen T.D. (2001). Analysis of relative gene expression data using real-time quantitative PCR and the 2^−ΔΔCT^ method. Methods.

