# Supplementary material for: Target Mechanism of Iturinic Lipopeptide on Differential Expression Patterns of Defense-Related Genes against Colletotrichum acutatum in Pepper
Source: Plants (Basel). 2022 May 9;11(9):1267. doi: 10.3390/plants11091267 (PMC9102045; doi:10.3390/plants11091267)
Supplement: Supplementary file 1 [file plants-11-01267-s001.zip › plants-1712534-supplementary.pdf]

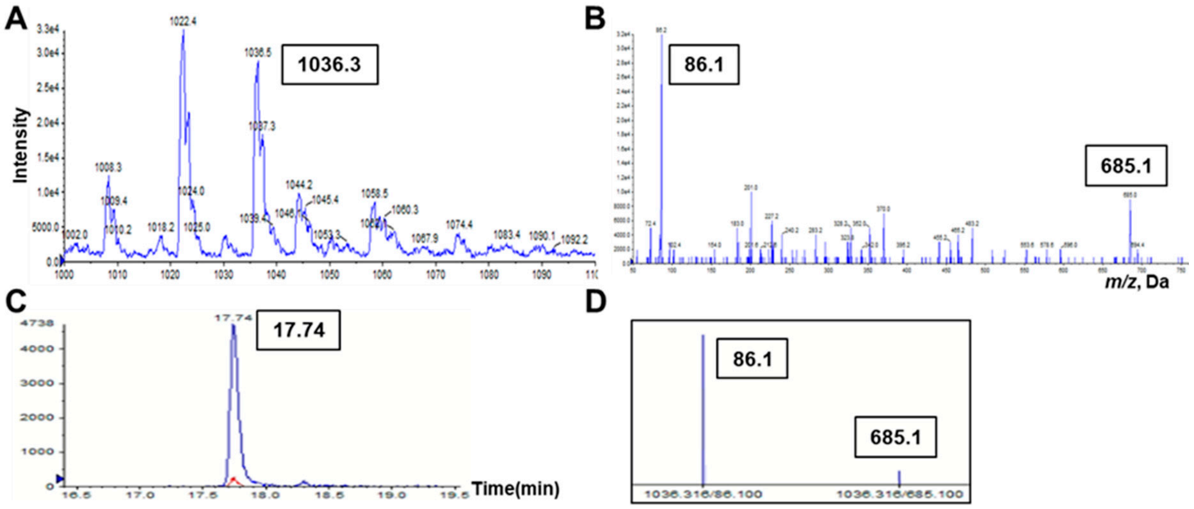

Supplementary Figure S1. Molecular ion peaks and mass spectra of surfactin of Bs KB21. (A) ESI-MS full scan MS (+) m/z corresponding to surfactin (B) Mass spectra corresponding to the peak m/z 1036.3 of surfactin (C) LC-MS/MS MRM chromatogram of surfactin standard at 0.05 mg/L (Retention time: 17.74 min) (D) Mass spectrum of surfactin (quantification ion: m/z 86.1 and qualification ion: m/z 685.1).

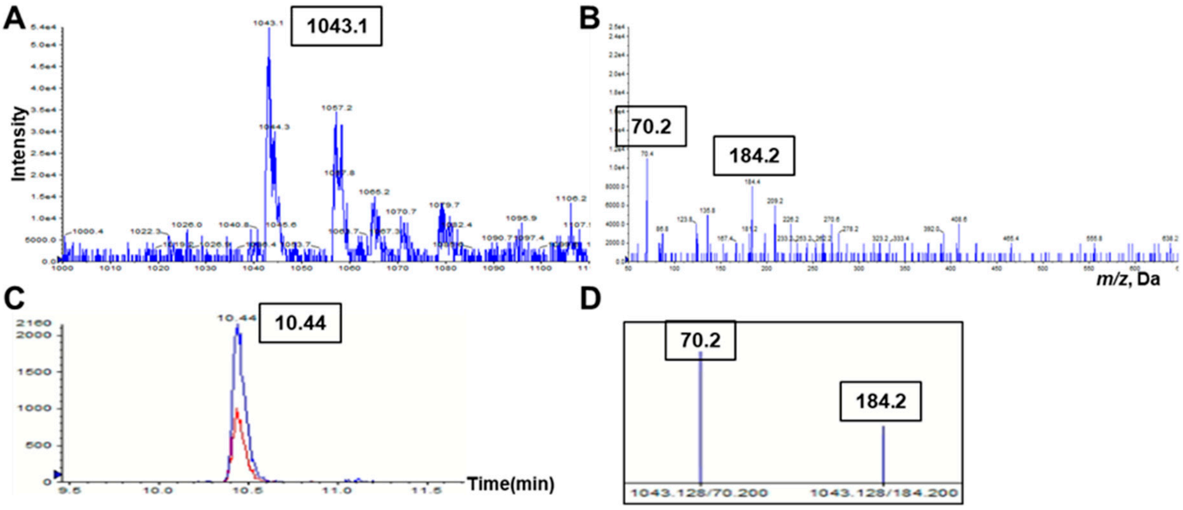

Supplementary Figure S2. Molecular ion peaks and mass spectra of iturin of Bs KB21. (A) ESI-MS full scan MS (+) m/z corresponding to iturin (B) Mass spectra corresponding to the peak m/z 1043.1 of iturin (C) LC-MS/MS MRM chromatogram of iturin A standard at 5 mg/L (Retention time: 10.44 min) (D) Mass spectrum of iturin (quantification ion: m/z 70.2 and qualification ion: m/z 184.2)

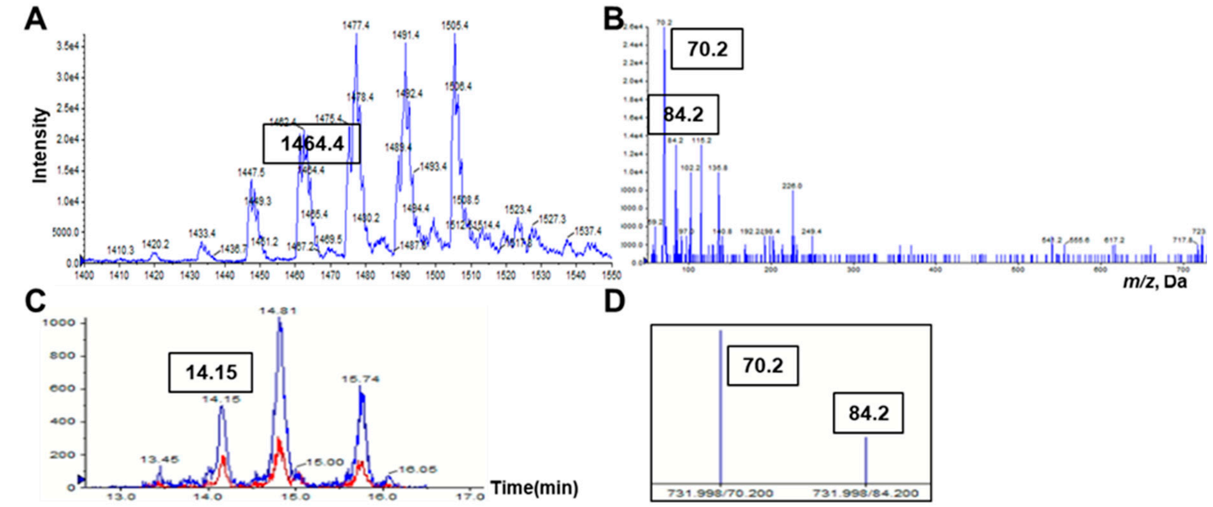

Supplementary Figure S3. Molecular ion peaks and mass spectra of fengycin of Bs KB21. (A) ESI-MS full scan MS (+) m/z corresponding to fengycin (B) Mass spectra corresponding to the peak m/z 1464.4 of fengycin (C) LC-MS/MS MRM chromatogram of fengycin standard at 5 mg/L (Retention time: 14.15 min) (D) Mass spectrum of fengycin (quantification ion: m/z 70.2 and qualification ion: m/z 84.2)

Supplementary Table S1

MRM transition parameters of analytes in LC-MS/MS

Compound	Precursor ion (m/z)	Product ion (m/z)	DP [b] (V)	EP [c] (V)	CE [d] (V)	Ionization mode
Iturin A	1043.47	70.1[a]	161.0	6.0	177.0	Positive ESI [e]
		183.9	161.0	14.0	107.0	
Surfactin	1036.59	86.0[a]	196.0	6.0	119.0	Positive ESI [e]
		685.5	196.0	14.0	45.0	
Fengycin	732.26	70.1	121.0	6.0	133.0	Positive ESI [e]
		84.0	121.0	14.0	121.0	

[a]Quantitation ion, [b]Declustering potential, [c]Entrance potential, [d]Collision energy [e]Electrospray ionization.
